# Characterization of microRNA candidates at the primary site of infectious bronchitis virus infection: A comparative study of *in vitro* and *in vivo* avian models

**DOI:** 10.1371/journal.pone.0319153

**Published:** 2025-03-11

**Authors:** Kelsey O’Dowd, Safieh Vatandour, Sadhiya S. Ahamed, Martine Boulianne, Charles M. Dozois, Carl A. Gagnon, Neda Barjesteh, Mohamed Faizal Abdul-Careem

**Affiliations:** 1 Health Research Innovation Centre, Faculty of Veterinary Medicine, University of Calgary, Calgary, Alberta, Canada; 2 Department of Animal and Poultry Science, Islamic Azad University, Qaemshahr Branch, Qaemshahr, Mazandaran, Iran; 3 Swine and Poultry Infectious Diseases Research Centre – Fonds de recherche du Québec (CRIPA-FRQ), Faculty of Veterinary Medicine, Université de Montréal, Saint-Hyacinthe, Québec, Canada; 4 Department of Clinical Sciences, Faculty of Veterinary Medicine, Université de Montréal, Saint-Hyacinthe, Québec, Canada; 5 Institut National de Recherche Scientifique-Centre Armand-Frappier Santé Biotechnologie, Laval, Québec, Canada; 6 Molecular Diagnostic and Virology Laboratories, Centre de diagnostic vétérinaire de l’Université de Montréal (CDVUM), Faculty of Veterinary Medicine, Université de Montréal, Saint-Hyacinthe, Québec, Canada; Keele University School of Life Sciences, UNITED KINGDOM OF GREAT BRITAIN AND NORTHERN IRELAND

## Abstract

Infectious bronchitis virus (IBV) is an important avian pathogen with a positive-sense single-stranded RNA genome. IBV is the causative agent of infectious bronchitis (IB), a primarily respiratory disease affecting chickens, with the ability to disseminate to other organ systems, such as the gastrointestinal, renal, lymphoid, and reproductive systems. Tracheal epithelial cells are the primary target of IBV, and these cells play a vital role in the effective induction of the antiviral response and eventual clearance of IBV. The host immune system is regulated by a number of different molecular players, including micro-ribonucleic acids (microRNAs), which are small, conserved, non-coding RNA molecules that regulate gene expression of complementary messenger RNA (mRNA) sequences, resulting in gene silencing through translational repression or target degradation. The goal of this study was to characterize and compare the microRNA expression profiles in chicken tracheal epithelial cells (cTECs) *in vitro* and the trachea *in vivo* upon IBV Delmarva/1639 (DMV/1639) or IBV Massachusetts 41 (Mass41) infections. We hypothesized that IBV infection influences the expression of the host microRNA expression profiles. cTECs and young specific pathogen-free (SPF) chickens were infected with IBV DMV/1639 or IBV Mass41 and the microRNA expression at 3 and 18 hours post-infection (hpi) in the cTECs and at 4 and 11 days post-infection (dpi) in the trachea were determined using small RNA-sequencing (RNA-seq). We found that the profile of differentially expressed (DE) microRNAs is largely dependent on the IBV strain and time point of sample collection. Furthermore, we predicted the interaction between host microRNA and IBV viral RNA using microRNA-RNA interaction prediction platforms. We identified several candidate microRNAs suitable for future functional studies, such as gga-miR-155, gga-miR-1388a, gga-miR-7/7b and gga-miR-21-5p. Characterizing the interaction between IBV and the host cells at the level of microRNA regulation provides further insight into the regulatory mechanisms involved in viral infection and host defense in chickens following IBV infection.

## Introduction

Infectious Bronchitis (IB) is primarily a respiratory disease in chickens caused by infection with infectious bronchitis virus (IBV), a *Gammacoronavirus* [1]. The clinical signs typically include gasping, coughing, tracheal rales and nasal discharge, and the virus can be spread by aerosol as well as by mechanical transmission [1]. Furthermore, IBV infection can predispose chickens to secondary bacterial infections, such as *Escherichia coli* infections, for example, exacerbating the respiratory clinical signs [2–4].

While IBV primarily targets respiratory epithelial cells, the virus can disseminate to other organs systems, such as the gastrointestinal, renal, lymphoid, and reproductive systems, and replicate at the epithelial surfaces of various tissues, depending on the strain [5,6]. The phenomenon that IBV infection in young chickens causes lasting damage to the reproductive tract, including the oviduct, leading to cystic oviduct and false layer chickens, has long been known [7]. Decreased egg production and quality have been associated with IBV strain Mass41 [8,9] and the more recent and increasingly prevalent IBV DMV/1639 strain [10–14]. Aside from standard biosecurity measures, live-attenuated and killed IB vaccines are the primary and most effective methods of control for IBV infections. However, these methods are limited in terms of cross-protection, as new variants of IBV frequently emerge due to selective pressure and to the nature of this highly mutable RNA virus [15]. Even after thorough investigation of different IBV strains, tissue tropisms, and shedding patterns, knowledge gaps persist regarding host antiviral responses at the primary site of infection. Considering this and the limitations of IBV vaccination, there is a need to gain a better understanding of host-pathogen interactions at the early stages of viral infection and to develop novel control strategies for IBV using immunostimulatory molecules or developing alternative vaccine adjuvants to increase vaccine efficacy.

For example, micro-ribonucleic acids (microRNAs) are known modulators of a variety of biological processes, including development, cancer, and infection. These RNA molecules are small, conserved, non-coding RNA molecules that regulate gene expression of complementary messenger RNA (mRNA) sequences, resulting in gene silencing through translational repression or target degradation [16,17]. The accumulating evidence over the last three decades of microRNA research has demonstrated the important role of these RNA molecules during infections across many host species. Certain microRNAs are modulated upon viral infection and can regulate the host antiviral response by targeting intracellular signalling pathways or by directly interacting with the virus to inhibit viral replication [18]. More specifically, avian microRNAs have been identified during infections with avian viruses, such as IBV [19,20], avian influenza virus (AIV) [21–23], infectious bursal disease virus (IBDV) [24,25], Marek’s disease virus (MDV) [26,27] and avian leukosis virus subgroup J (ALV-J) [28,29], which are reviewed in detail by Duan et al. (2020) and Wang (2020) [30,31]. For example, gga-miR-26a was found to regulate melanoma differentiation-associated protein 5 (MDA5) during highly pathogenic AIV infection by targeting its 3’ untranslated region (UTR) [22]. This same microRNA was also found to target never in mitosis gene A (NIMA)-related kinase-6 (NEK6) and suppress Marek’s disease lymphoma cell proliferation [26]. Another important avian microRNA is gga-miR-155, which was shown to enhance type I IFN expression in a chicken fibroblast cell line (DF-1) following IBDV infection by targeting suppressor of cytokine signaling 1 (SOCS1) and tumor necrosis factor (TNF) receptor-associated factor (TRAF) family member-associated nuclear factor-κB (TANK) [24]. Moreover, the results from our previous study show that specific microRNAs expressed in chicken tracheal cells following AIV infection have the potential to target segments of the AIV genome directly and elicit critical pathways in the antiviral responses [32].

In the context of IBV infections, *in vitro* studies in a chicken macrophage cell line, HD11, determined that gga-miR-30d inhibited IBV replication by targeting ubiquitin-specific peptidase 47 (USP47) [20] and gga-miR-146a enhanced IBV replication by targeting interleukin-1 receptor-associated kinase 2 (IRAK2) and TNF receptor superfamily member 18 (TNFRSF18) [19]. In addition, studies profiling microRNA upon IBV infection have been conducted in chicken kidneys [33], avian bone marrow-derived dendritic cells (BMDCs) [34,35] and the spleen and lungs of chicken embryos [36]; however, no profiling studies evaluating the microRNA contents of IBV-infected tracheal cells or the trachea have been conducted.

Our previous study characterized the mRNA expression profiles using RNA-seq in chicken tracheal epithelial cells (cTECs) and the chicken trachea infected with two prevalent IBV strains, DMV/1639 and Mass41, and identified key differentially expressed genes involved in the antiviral responses for both the *in vitro* and *in vivo* infection models [37]. In the present study, we aimed to study the response to IBV infection at the level of microRNA regulation in these same samples to evaluate the impact of microRNA expression using small RNA-seq. We hypothesized that infection of chicken tracheal cells or tissues with IBV influences the host microRNA expression profiles and that certain microRNAs expressed in the respiratory tract may be crucial in regulating the antiviral response or IBV replication. By screening these potentially regulatory avian microRNAs, we identify candidates suitable for future functional studies *in vitro* and *in vivo* which may have important implications in immune response signalling.

## Materials and methods

### Experimental design

Detailed information for the virus propagation and titration, cTEC preparation and IBV infection *in vitro*, animals (chickens) and IBV infection *in vivo*, can be found in our previous study O’Dowd et al. (2024) [37]. For the *in vitro* experiments, an early time point, 3 h, and at a later time point near the peak of viral genome load detected, 18 h, were selected [37]. For the *in vivo* experiments, since replication of IBV in the respiratory tract usually peaks between 3 and 7 dpi [38,39], 4 dpi was selected as a time point at the peak of replication and 11 dpi was selected as a time point later on during infection. Briefly, the Canadian IBV DMV/1639 clinical isolate IBV/Ck/Can/17-036989 (GenBank accession no. MN512435), and the Canadian IBV Mass41 clinical isolate IBV/Ck/Can/21-2455844 (GenBank accession no. PP373115) were used for infection of primary cTECs and specific-pathogen-free (SPF) chickens (layer chickens, white Leghorn) obtained from the Canadian Food Inspection Agency (CFIA), Ottawa, ON, Canada.

For IBV infection *in vitro*, the cTECs were prepared based on previously established protocols, with some modifications [40–42]. The cTECs were isolated from the tracheas of 19-day-old SPF chicken embryos and cultured in complete Dulbecco’s Modified Eagle Medium/Nutrient Mixture F-12 (DMEM/F-12, Gibco, Burlington, ON, Canada) supplemented with 10% chicken embryo extract, which was prepared in-house, as previously described [43]. The cTECs were subsequently infected with 1 × 10^5^ EID_50_/mL in 200 µ L of either IBV DMV/1639 or IBV Mass41 in complete DMEM/F-12 (serum-free) infection media, washed twice at 2 hours post-infection (hpi), and incubated in fresh media. The uninfected control group received media only. The cells were collected in QIAzol® reagent (QIAGEN, Toronto, ON, Canada) at 3 h and 18 h for small RNA sequencing.

For IBV infection *in vivo*, one-day-old specific-pathogen-free (SPF) embryonated chickens (layer chickens, white Leghorn) (n =  60) from the CFIA, Ottawa, ON, and housed in the animal facilities at the National Experimental Biology Laboratory (NEBL) of the Institut national de la recherche scientifique (INRS) Armand-Frappier Santé Biotechnologie Research Centre. The chickens were randomly divided into 5 groups (n =  12 chickens/group): IBV DMV/1639 low dose, IBV DMV/1639 high dose, IBV Mass41 low dose, IBV Mass41 high dose and uninfected control. The number of animals used for the experiment was selected following consultation with a biostatistician. The experimental protocols were approved by the Institutional Animal Care and Use Committee (IACUC) of the Université de Montréal (ethics protocol no. 21-Rech-2120) and the INRS (ethics protocol no. 2106-03). At 6 days of age, SPF chickens were inoculated with 100 µ L of either IBV DMV/1639 or IBV Mass41 containing 10^4^ EID_50_/bird or 10^5^ EID_50_/bird through the intranasal and intraocular routes. The negative control group received phosphate-buffered saline (PBS).

Chickens were monitored for possible clinical signs, including depression, coughing, sneezing, ruffled feathers, wet droppings, tracheal rales, conjunctivitis, dyspnea and facial swelling. Within 24 hours, in the case of no progress/improvement in affected chickens and worsened conditions with symptoms including severe respiratory distress, wet droppings, and unconsciousness that causes distress and difficulty in breathing, affected chickens should be euthanized. No animals died before meeting the criteria for euthanasia.

The chickens were monitored twice daily in the first 24 hpi and daily after the first 24 hpi. In addition to the continuous monitoring of animals before and after infection for health and behavior changes to determine baseline status and humane endpoints (including decreased mobility interfering with the basic activity of the chickens, reduced activity, severe respiratory distress, wet droppings, unconsciousness), chicken isolators were used, and a maximum 15 chickens were kept in each isolator. All research staff were trained for mandatory biosecurity and animal handling training at the National Experimental Biology Centre (CNBE) of the Université du Québec - Institut national de la recherche scientifique (INRS), Laval, Québec.

The experiment duration was 11 days. At 4 (n =  6 chickens/group) and 11 dpi (n =  6 chickens/group), oropharyngeal (OP) and cloacal (CL) swabs were taken and birds were euthanized by CO_2_ administration followed by cervical dislocation before samples from the upper half of the trachea were collected. The swabs were kept in virus transport medium (Puritan® UniTranz-RT® Universal Transport Solution, VWR, Montreal, QC, Canada) and the tissue samples were kept in RNAlater® (Invitrogen, Burlington, ON, Canada) for downstream IBV genome load and small RNA-seq analysis.

### Quantification of IBV genome load and validation of host microRNA expression

The OP and CL swabs collected during the *in vivo* experiments were lysed in TRIzol™ LS reagent (Invitrogen, Burlington, ON, Canada). Total RNA was extracted according to the manufacturer’s protocol and isolated RNA was resuspended in 20 µ L RNase-free water. Complementary deoxyribonucleic acid (cDNA) synthesis was performed for 500 ng of RNA per sample using the High-Capacity Reverse Transcription Kit with random primers (Applied Biosystems, Waltham, MA, USA) according to manufacturer’s instructions.

Using IBV-N gene-specific forward 5′GACGGAGGACCTGATGGTAA-3′ and reverse 5′CCCTTCTTCTGCTGATCCTG-3′ primers at a final concentration of 5 nM (Sigma-Aldrich, Saint-Louis, MO, USA) and PowerUp SYBR Green Master Mix (Applied Biosystems, Burlington, ON, Canada) in a 20 µ L reaction according to the manufacturer’s instructions, as previously described [44], real-time quantitative polymerase chain reaction (qPCR) targeting the IBV nucleocapsid gene (N) was performed for quantification of IBV genome load in the OP and CL swabs. The qPCR cycling program was as follows: pre-incubation at 95 °C for 20 seconds (sec), and amplification/extension at 95 °C for 3 sec and 60 °C for 30 sec, repeated for 40 cycles. Melting curve analysis was assessed at 95 °C for 10 sec (segment 1), 65 °C for 5 sec (segment 2) and 9 °C for 5 sec (segment 3), and fluorescence acquisition was done at 60 °C for 30 sec. A 10-fold dilution series of a IBV-N gene plasmid standard was used to produce a standard curve [44] and results for IBV genome load are presented as log_10_ IBV genome copies per 1 μL of reaction/cDNA.

RNA was isolation for the tracheal samples is described in our previous study O’Dowd et al. (2024) [37]. The cDNA was synthesized from 10 ng of RNA using the TaqMan MicroRNA Reverse Transcription kit (Applied Biosystems, Burlington, ON, Canada) and the qPCR was performed using the TaqMan Universal Master Mix II (Applied Biosystems, Burlington, ON, Canada), according to manufacturer’s instructions. Fold-change (FC) was calculated using the 2^-ΔΔCt^ method [45]. The following TaqMan primer and probe sets were used: dre-miR-187 (assay ID 007816_mat), gga-miR-458 * (assay ID 006129_mat), oan-miR-1388 * (assay ID 007800_mat), hsa-miR-155 (assay ID 000479) and U6 small nuclear (sn)RNA (assay ID 001973) (Thermo Fisher Scientific, Massachusetts, USA).

### RNA isolation, cDNA library preparations and high throughput sequencing

Detailed information about the samples used and for RNA isolation is described in our previous study O’Dowd et al. (2024) [37]. cTEC small RNA library preparations and sequencing were done at the Plateforme de séquençage de nouvelle génération of the University Research Center of the CHU de Québec-Université Laval, Quebec, Canada following RNA isolation. Twenty-four libraries were prepared for small RNA-sequencing (RNA-seq): IBV DMV/1639 3 h, IBV DMV/1639 18 h, IBV Mass41 3 h, IBV Mass 18 h, control (CTRL) 3 h, CTRL 18 h. Infected samples are from cTECs infected with a high dose of 5 × 10^5^ 50% embryo infectious dose (EID_50_)/mL of IBV. Tracheal small RNA library preparations and sequencing were done at the McGill Applied Genomics Innovation Core (MAGIC) of the McGill Genome Centre, McGill University, Montreal, Quebec, Canada. Eighteen libraries were prepared for small RNA-seq: IBV DMV/1639 4 dpi, IBV DMV/1639 11 dpi, IBV Mass41 4 dpi, IBV Mass41 11 dpi, CTRL 4 dpi, CTRL 11 dpi. The tracheal samples used for sequencing were from tissues that originate from chickens infected with a high dose (10^5^ EID_50_/bird) of IBV. The small RNA libraires were sequenced on a NovaSeq 6000 S4 (Illumina, San Diego, CA, USA) platform which generated 100 base pair (bp) paired-end reads.

### Small RNA-seq differential expression analysis

Data analysis for small RNA-seq data was done using the open-source framework, GenPipes [46] and conducted using RStudio, unless stated otherwise [47,48]. For analysis and formatting of the data and results, the R packages knitr [49], ggrepel [50], tibble [51], tidyverse [52], magrittr [53], hablar [54] and kableExtra [55] were used. The miRDeep2 software was used for small RNA-seq data analysis [56–58]. Quality controlled data obtained after processing raw reads using Bowtie 1 [59], Cutadapt [60] and FastQC [61] were visualized in MultiQC [62]. The resulting reads were mapped to the chicken reference genome and the database of known chicken microRNAs (precursors, mature and hairpin) available in miRBase version 22.1 [63,64] using the miRDeep2 software [56–58] mapper module and quantified using the quantifier module, which counts reads for known microRNAs in the dataset. Read signatures, scores and statistics are generated and reported for each microRNA. The differential expression analysis was then performed using the DESeq2 R package [65,66], as described for the RNA-seq data analysis in our previous study [37], and the data was batched normalized and log transformed. Differential microRNA expression is based on an infected group compared to the uninfected control group at the same time point. MicroRNAs are considered differentially expressed (DE) if the adjusted p-value <  0.05 and log_2_FC ≥  | 0.58 | (FC ≥  | 1.5|). Principle Component Analysis (PCA) plots, heatmaps using R packages ComplexHeatmap [67] and tidyHeatmap [68], and volcano plots using the R package EnhancedVolcano [69], were created in R [47,48]. Venn diagrams were created using the online tools https://bioinformatics.psb.ugent.be/webtools/Venn/ and Venny [70]. The R package miRBaseConverter [71] was used to access microRNA accession and name information.

In addition, the miRDeep2 software [56–58] was used to scan the small RNA-seq data for novel microRNAs based on defined microRNA properties and miRBase version 22.1 [63,64]. Identified microRNAs were considered novel microRNAs if the miRDeep2 score >  4 [72,73].

### Target gene prediction, gene ontology (GO) and pathway analysis for targets of DE microRNAs

The microRNA target prediction algorithm miRDB version 6.0 (using the miRtarget4 algorithm) [74,75] was used to determine host gene targets of the DE microRNAs. The target mining function was used to search for microRNAs for gene targets, excluding gene targets with target prediction score <  60 and number of microRNA predicted targets in the genome >  2000. The species was set to “Chicken” (*Gallus gallus*). GO functional enrichment analyses, or over-representation analyses (ORA), and visualization for Biological Process (BP), Molecular Function (MF) and Cellular Component (CC) were performed for targets of down- and up-regulated microRNAs using the R packages gprofiler2 (g:Profiler) [76,77] with p-values (threshold of α = 0.05) based on a hypergeometric test and multiple test correction using the g:GOSt with default g:SCS method, enrichplot [78], DOSE [79] and ggplot2 [80]. The parameters used were as follows: the set of known genes was used as background and terms with GO evidence codes Inferred from Electronic Annotation (IEA) were excluded. The R package GOfuncR was used to assess the relation between GO term parent and child nodes [81]. The R packages gprofiler2 (g:Profiler) [76,77], pathview [82] and org.Gg.eg.db [83] were used to perform Kyoto Encyclopedia of Genes and Genomes (KEGG) [84] pathway analysis and visualization.

### IBV viral target of host DE microRNAs

The microRNA-RNA interaction platforms, miRDB version 6.0 (using the miRtarget4 algorithm) [74,75], miRanda [85] and RNAhybrid [86] were used to scan the IBV DMV/1639 clinical isolate IBV/Ck/Can/17-036989 (GenBank accession no. MN512435) and the IBV Mass41 isolate IBV/Ck/Can/21-2445844 (GenBank accession no. PP373115) whole genome sequences for potential target site of the DE microRNAs. For miRDB, custom target prediction for “Chicken” (*Gallus gallus*) microRNAs was performed and results were filtered for scores >  95. The source code for miRanda was downloaded from http://www.microrna.org/microrna/getDownloads.do and was used with default parameters for scaling parameter (4.0), strict 5’ seed pairing (off), gap-opening penalty (−4.0), and gap-extend penalty (−9.0), and adjusted parameters for score sc ≥  160 and the minimum free energy (mfe) en ≤  − 16 kcal/mol) [87,88]. RNAhybrid [86] was then used to assess microRNA-RNA interactions and hybridization. The figure for viral targets of DE microRNAs was created using Geneious version 2023.2 created by Biomatters, available from https://www.geneious.com.

### Statistical analysis

The statistical methods for small RNA sequencing data analysis are contained within the software used. For IBV genome loads, differences were assessed using three-way analysis of variance (ANOVA) followed by Tukey’s post hoc test. The differences were considered significant if the p-value <  0.05 and statistical analysis was performed using GraphPad Prism 10 software (GraphPad, La Jolla, CA, USA).

## Results

### IBV infection

#### IBV infection in cTECs.

Evidence of establishment of IBV infection in cTECs was determined via quantification of IBV genome load in cTECs and published previously [37].

#### IBV infection in chickens.

**Fig 1:**
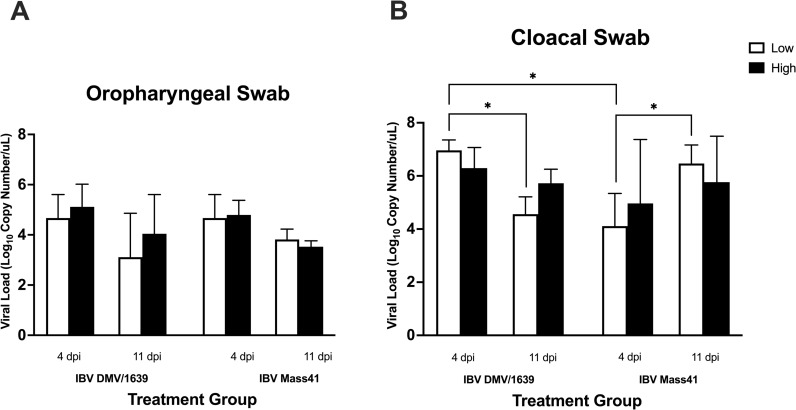
IBV genome shedding from chickens infected with IBV DMV/1639 or IBV Mass41. Chickens were infected with a low (10^4^ EID_50_/bird) or a high (10^5^ EID_50_/bird) dose of either IBV DMV/1639 or IBV Mass41, and at 4 dpi and 11 dpi, **(A)** OP swabs from chickens infected with IBV DMV/1639 or IBV Mass41 and **(B)** CL swabs from chickens infected with IBV DMV/1639 or IBV Mass41 were collected to determine viral genome load using qPCR. Statistical analysis for differences in IBV genome loads was assessed using three-way ANOVA followed by Tukey’s post hoc test. Significant differences (p-value <  0.05) are denoted by * . The error bars represent the standard deviation (SD).

The presence of IBV genome was assessed in swabs from chickens infected with a low (10^4^ EID_50_/bird) or a high (10^5^ EID_50_/bird) dose of IBV DMV/1639 or IBV Mass41 at 4 dpi and 11 dpi. Swab samples from all challenged groups were IBV-positive. IBV RNAs (genomic and subgenomic) were not detected in uninfected controls at 4 dpi and 11 dpi. OP and CL shedding are shown in Fig 1. For the OP swabs from the challenged groups, there were no significant differences (p-value >  0.05) observed among the different strains, doses and time points (Fig 1A). On the other hand, for the CL swabs, a significant decrease (p-value <  0.05) in IBV genome load was observed from 4 dpi to 11 dpi for the IBV DMV/1639 low dose groups (Fig 1B). There was a significant increase (p-value <  0.05) in CL IBV genome load from 4 dpi to 11 dpi for the IBV Mass41 low dose groups. The different patterns for the CL shedding for the low dose groups are opposite, where IBV DMV/1639-infected group’s shedding decreases and IBV Mass41-infected group’s shedding increases over time and this may be due to differential replication dynamics here. Furthermore, CL shedding was significantly higher (p-value <  0.05) in the low dose IBV DMV/1639-infected group as compared to the low dose IBV Mass41-infected group at 4 dpi.

Previously, we reported the IBV genome loads in the tracheas of IBV-infected chickens from this experiment [37].

### MicroRNA expression patterns of cTECs infected with different IBV strains

**Fig 2:**
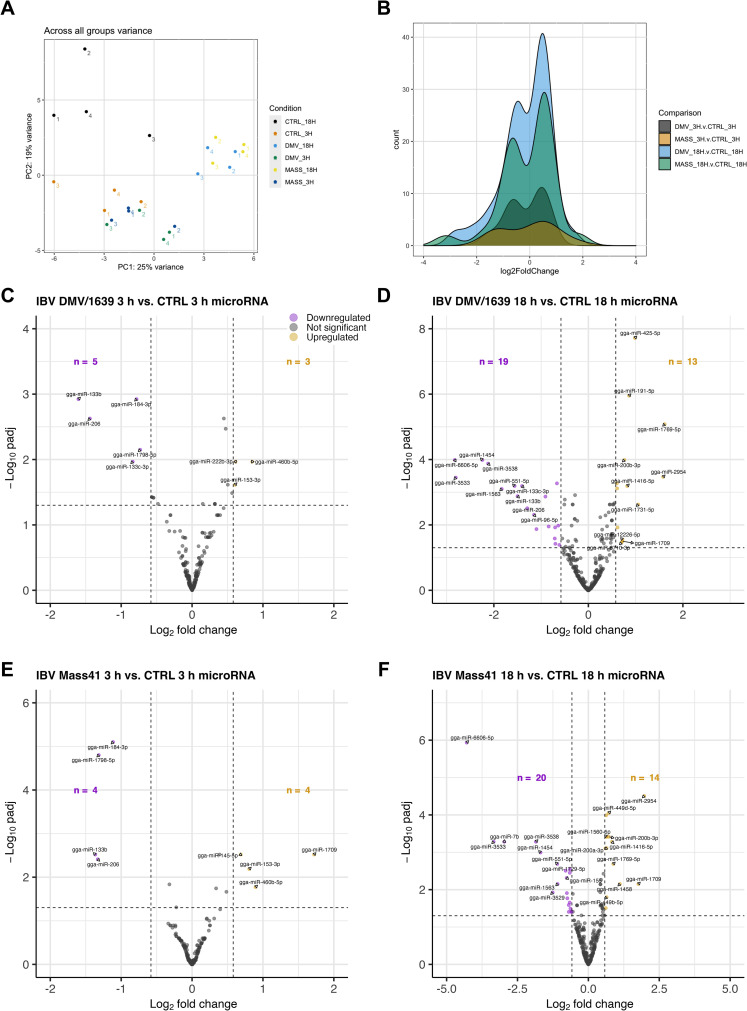
MicroRNA expression in cTECs infected with IBV DMV/1639 or IBV Mass41. Panel (A) shows the variance across all samples based on the log counts of all microRNAs using a PCA plot. The histogram in panel (B) represents the log_2_FC distribution of fluorescence signal intensity ratios for DE microRNAs of cTECs infected with IBV DMV/1639 or IBV Mass41 at 3 h and 18 h. The volcano plots present DE microRNAs of cTECs infected with IBV DMV/1639 at 3 **h** (C) and 18 **h** (D) or IBV Mass41 at 3 **h** (E) and 18 **h** (F), relative to the respective uninfected control groups. The horizontal dotted line marks the threshold for the adjusted p-value <  0.05 and the vertical dotted lines denote the log_2_FC ≥  | 0.58 | (FC ≥  | 1.5|) threshold. Down-regulated microRNAs are indicated by purple data points and up-regulated microRNAs are indicated by yellow data points. A summary of all up- and down-regulated microRNAs for each treatment group are provided in Table 1.

Small RNA-seq differential expression results for cTECs are compiled in S1 Table and results filtered for significant differences (defined by an adjusted p-value <  0.05 and a log_2_FC ≥  | 0.58|) are compiled in Table 1. Fig 2A shows the variance in log counts across all samples by group. The heatmaps (S1 Fig) show the differences between cTECs infected with IBV DMV/1639 at 3 h (S1A Fig) and 18 h (S1B Fig) or IBV Mass41 at 3 h (S1C Fig) and 18 h (S1D Fig), relative to the respective control groups. These data show the differences in counts between the IBV-inoculated cTECs and uninfected control cTECs for the microRNA expression data.

**Table 1:** DE microRNAs from cTECs infected with IBV DMV/1639 or IBV Mass41.

Treatment Group		Mature MicroRNA	Log_2_FC	Adjusted P-Value
**IBV DMV/1639 3 h**	Down-regulated (5)	gga-miR-133b	-1.603	1.194E-03
		gga-miR-206	-1.441	2.360E-03
		gga-miR-133c-3p	-0.837	1.079E-02
		gga-miR-184-3p	-0.783	1.194E-03
		gga-miR-1798-5p	-0.732	7.093E-03
	Up-regulated (3)	gga-miR-153-3p	0.606	2.427E-02
		gga-miR-222b-3p	0.613	1.079E-02
		gga-miR-460b-5p	0.849	1.079E-02
**IBV DMV/1639 18 h**	Down-regulated (19)	gga-miR-6606-5p	-2.827	1.026E-04
		gga-miR-3533	-2.805	3.564E-04
		gga-miR-1454	-2.243	1.026E-04
		gga-miR-3538	-2.122	1.326E-04
		gga-miR-1563	-1.833	7.953E-04
		gga-miR-551-5p	-1.561	6.446E-04
		gga-miR-133b	-1.499	1.322E-03
		gga-miR-133c-3p	-1.404	6.446E-04
		gga-miR-206	-1.294	3.091E-03
		gga-miR-96-5p	-1.150	4.911E-03
		gga-miR-7b	-1.099	1.344E-02
		gga-miR-1729-5p	-0.909	1.362E-03
		gga-miR-12288-5p	-0.840	1.118E-02
		gga-miR-199b	-0.711	2.603E-02
		gga-miR-133a-3p	-0.704	1.197E-02
		gga-miR-6659-3p	-0.698	3.786E-02
		gga-miR-15c-5p	-0.665	5.358E-04
		gga-miR-214	-0.637	1.050E-02
		gga-miR-7475-5p	-0.613	4.090E-02
	Up-regulated (13)	gga-miR-449d-5p	0.605	5.358E-04
		gga-miR-200a-3p	0.609	7.641E-04
		gga-miR-449b-5p	0.617	1.197E-02
		gga-miR-6710-3p	0.699	3.519E-02
		gga-miR-1709	0.720	3.158E-02
		gga-miR-12226-5p	0.722	2.800E-02
		gga-miR-200b-3p	0.762	1.026E-04
		gga-miR-1416-5p	0.822	6.446E-04
		gga-miR-191-5p	0.860	1.124E-06
		gga-miR-425-5p	0.984	1.920E-08
		gga-miR-1731-5p	1.054	2.375E-03
		gga-miR-2954	1.582	3.385E-04
		gga-miR-1769-5p	1.614	8.313E-06
**IBV Mass41 3 h**	Down-regulated (4)	gga-miR-133b	-1.365	3.021E-03
		gga-miR-206	-1.330	3.879E-03
		gga-miR-1798-5p	-1.316	1.582E-05
		gga-miR-184-3p	-1.114	7.929E-06
	Up-regulated (4)	gga-miR-145-5p	0.687	3.021E-03
		gga-miR-153-3p	0.806	6.494E-03
		gga-miR-460b-5p	0.895	1.689E-02
		gga-miR-1709	1.721	3.021E-03
**IBV Mass41 18 h**	Down-regulated (20)	gga-miR-6606-5p	-4.292	1.162E-06
		gga-miR-3533	-3.340	5.218E-04
		gga-miR-7b	-2.966	5.218E-04
		gga-miR-3538	-1.851	5.218E-04
		gga-miR-1454	-1.687	1.012E-03
		gga-miR-3529	-1.259	1.191E-02
		gga-miR-551-5p	-1.096	2.053E-03
		gga-miR-1563	-1.085	7.063E-03
		gga-miR-1729-5p	-0.802	3.117E-03
		gga-miR-155	-0.764	4.840E-03
		gga-miR-133c-3p	-0.759	1.237E-02
		gga-miR-451	-0.741	1.707E-02
		gga-miR-96-5p	-0.693	2.580E-02
		gga-miR-7475-5p	-0.690	3.929E-02
		gga-miR-133a-3p	-0.659	2.212E-02
		gga-miR-184-3p	-0.656	3.543E-03
		gga-miR-147	-0.654	2.840E-03
		gga-miR-133b	-0.631	3.196E-02
		gga-miR-199b	-0.598	3.929E-02
		gga-miR-6631-5p	-0.594	3.929E-02
	Up-regulated (14)	gga-miR-191-5p	0.604	7.839E-04
		gga-miR-1731-5p	0.614	3.119E-02
		gga-miR-2131-5p	0.626	1.022E-04
		gga-miR-425-5p	0.640	3.845E-04
		gga-miR-1560-5p	0.642	3.845E-04
		gga-miR-200a-3p	0.642	7.741E-04
		gga-miR-449b-5p	0.643	1.592E-02
		gga-miR-449d-5p	0.720	8.865E-05
		gga-miR-200b-3p	0.722	3.845E-04
		gga-miR-1416-5p	0.831	5.218E-04
		gga-miR-1769-5p	0.878	2.053E-03
		gga-miR-1458	1.111	7.063E-03
		gga-miR-1709	1.763	7.063E-03
		gga-miR-2954	1.971	3.096E-05

**Fig 3:**
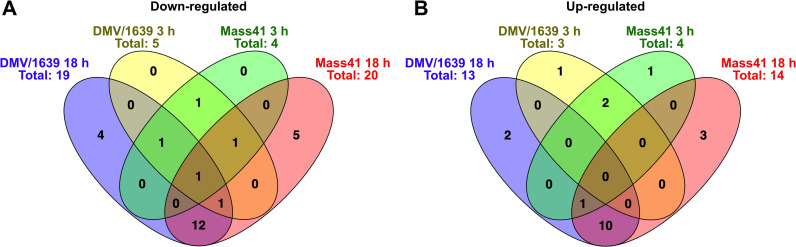
Overlap for DE microRNAs of cTECs infected with IBV DMV/1639 or IBV Mass41. The overlap is illustrated by Venn diagrams for down-regulated **(A)** and up-regulated **(B)** DE microRNAs of cTECs infected with IBV DMV/1639 or IBV Mass41 at 3 h and 18 h. The common and unique DE microRNAs are summarised in S2 Table.

There are a higher number of DE microRNAs at 18 h when compared to the 3 h groups for both IBV strains (Fig 2B). Among all IBV-inoculated groups, a total of 46 DE microRNAs were identified (Table 1), with 26 down-regulated and 20 up-regulated microRNAs. Fig 2C–F shows the number of down- and up-regulated microRNAs per group. Briefly, a total of 8 and 32 DE microRNAs, 5 and 19 down-regulated microRNAs, and 3 and 13 up-regulated microRNAs were identified for IBV DMV/1639 3 h and IBV DMV/1639 18 h, respectively. Furthermore, 8 and 34 DE microRNAs, 4 and 20 down-regulated microRNAs, and 4 and 14 up-regulated microRNAs were identified for IBV Mass41 3 h and IBV Mass41 18 h, respectively (S2 Table). There is a similar number of down- and up-regulated microRNAs within each group. Fig 3 shows the DE microRNAs present in more than one treatment group for down-regulated microRNAs (Fig 3A) and for up-regulated microRNAs (Fig 3B).

One microRNA was commonly down-regulated among all groups. For each of the following intersections, one microRNA was found to be down-regulated: IBV DMV/1639 18 h, IBV DMV/1639 3 h, and IBV Mass41 3 h; IBV DMV/1639 18 h, IBV DMV/1639 3 h, and IBV Mass41 18 h; and IBV DMV/1639 3 h, IBV Mass41 18 h, and IBV Mass41 3 h. One microRNA in both IBV strain groups at 3 h and 12 microRNAs in both IBV strain groups at 18 h were commonly down-regulated. Finally, 4 down-regulated microRNAs were found in the IBV DMV/1639 18 h group only and 5 down-regulated microRNAs were found in the IBV Mass41 group only. No microRNAs were up-regulated among all groups. One microRNA was up-regulated in the IBV DMV/1639 18 h, IBV Mass41 18 h, and IBV Mass41 3 h groups. Two microRNAs in both IBV strain groups at 3 h and 10 microRNAs in both IBV strain groups at 18 h were commonly up-regulated. Finally, 1, 2, 1, and 3 microRNAs were up-regulated in the IBV DMV/1639 3 h, IBV DMV/1639 18 h, IBV Mass41 3 h, and IBV Mass41 18 h groups, respectively.

Our results show that the microRNA expression profiles depend on the time point of collection. Few microRNAs are common between time points for each strain. No microRNAs are exclusively shared between the same IBV strain at different time points for both IBV DMV/1639 and IBV Mass41. For the down-regulated microRNAs, gga-miR-133b, was commonly down-regulated among all groups, while gga-miR-206, gga-miR-133c-3p, and gga-miR-184-3p were each down-regulated in 3 out of the 4 treatment groups. Furthermore, gga-miR-1709 was up-regulated in the IBV DMV/1639 3 h, IBV DMV/1639 18 h, and IBV Mass41 18 h groups. The majority of overlapping DE microRNAs are observed for the different strains at the same time point. The microRNA, gga-miR-1798, was down-regulated in the IBV DMV/1639 3 h and IBV Mass41 3 h groups, and gga-miR-96-5p, gga-miR-3538, gga-miR-1729-5p, gga-miR-3533, gga-miR-133a-3p, gga-miR-551-5p, gga-miR-1454, gga-miR-1563, gga-miR-7475-5p, gga-miR-6606-5p, gga-miR-199b, gga-miR-7b were down-regulated in the IBV DMV/1639 18 h and IBV Mass41 18 h groups. Gga-miR-460b-5p and gga-miR-153-3p were commonly up-regulated at the earlier time point for both strains, while gga-miR-200a-3p, gga-miR-200b-3p, gga-miR-191-5p, gga-miR-449b-5p, gga-miR-449d-5p, gga-miR-1731-5p, gga-miR-2954, gga-miR-1769-5p, gga-miR-425-5p, gga-miR-1416-5p were commonly up-regulated at the later time point. On the other hand, the microRNA expression profiles between strains were not identical. Four microRNAs, including gga-miR-214 and gga-miR-15c-5p, and 5 microRNAs, including gga-miR-155 and gga-miR-147, were uniquely down-regulated in the IBV DMV/1639 18 h group and the IBV Mass41 18 h group, respectively. Gga-miR-222b-3p was up-regulated in the IBV DMV/1639 3 h group only, gga-miR-6710-3p and gga-miR-12226-5p were up-regulated in the IBV DMV/1639 18 h group only, gga-miR-145-5p was up-regulated in the IBV Mass41 3 h group only, and gga-miR-1560-5p, gga-miR-1458 and gga-miR-2131-5p were up-regulated in the IBV Mass41 18 h group only.

In addition, the miRDeep2 software identified microRNAs considered novel, which revealed 332 predicted novel microRNAs with miRDeep2 score >  4. The details for the miRDeep2 analysis for novel microRNAs in the trachea small RNA-seq data are given in S3 Table.

### Target gene prediction of DE microRNAs from cTECs infected with different IBV strains

**Fig 4:**
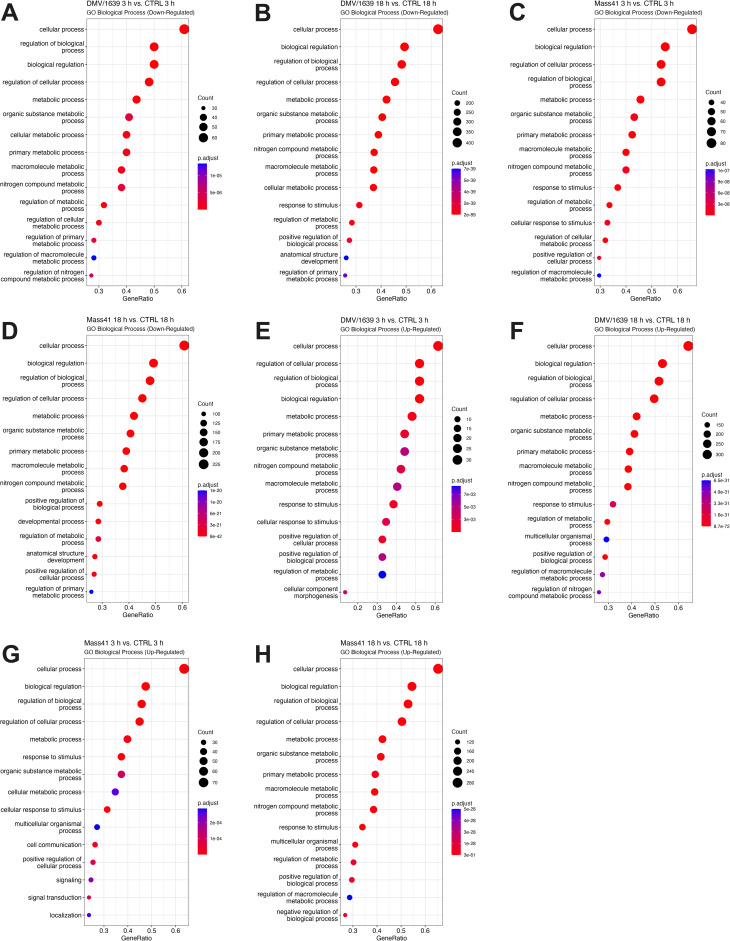
Functional analysis for targets of DE microRNAs from cTECs infected with IBV DMV/1639 or IBV Mass41. The dot plots illustrate the enriched GO (BP) terms for down-regulated target of DE microRNAs from the IBV DMV/1639 at 3 **h** **(A)**, IBV DMV/1639 at 18 **h** **(B)**, IBV Mass41 at 3 **h** **(C)**, and IBV Mass41 at 18 **h** (D) groups, and for up-regulated targets of DE microRNAs from the IBV DMV/1639 at 3 **h** **(E)**, IBV DMV/1639 at 18 **h** **(F)**, IBV Mass41 at 3 **h** **(G)**, and IBV Mass41 at 18 **h** (H) groups. The “Count” indicates the number of genes enriched in a GO term, while the “GeneRatio” reflects the percentage of total target of DE microRNAs in a specific GO term. The intensity of color corresponds to the adjusted p-values. A summary of all GO terms for targets of DE microRNAs can be found in S5 Table.

Target gene prediction of microRNAs by miRDB [74,75] revealed at least 50 targets per group (up- and down-regulated) with a score of > 95 (S4 Table). For example, DEAD-box helicase 3 X-linked (DDX3X) and helicase with zinc finger 2 (HELZ2) were both predicted targets of gga-miR-133b, a microRNA that was down-regulated in all cTEC treatment groups. The cut-off score of 95 was used to evaluate enriched GO and KEGG terms associated with these targets of DE microRNAs. Fig 4 shows the enriched GO terms (Biological Process) for targets of the DE microRNAs in the different treatment groups. Full details for GO and KEGG enrichment analysis are given in S5 Table. As there are many predicted targets for the DE microRNA, the top GO terms associated with the up- and down-regulated microRNA targets tend to be more associated with response to stimulus and metabolism.

**Fig 5:**
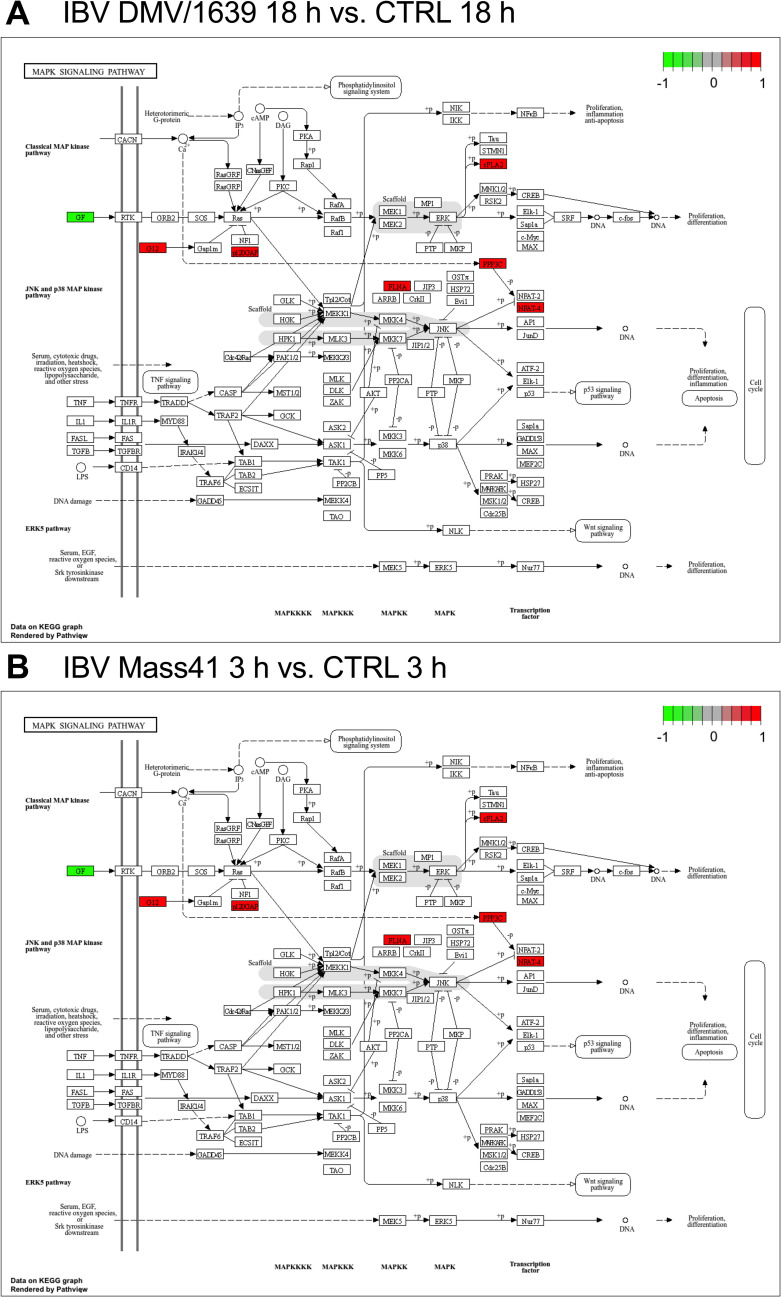
Pathway analysis for targets of DE microRNAs in cTECs infected with IBV DMV/1639 or IBV Mass41. The enrichment of pathways for IBV DMV/1639 18 **h** (A) and IBV Mass41 3 **h** **(B)** MAPK signalling is shown above. These KEGG pathway analysis figures were generated using the R package pathview. The intensity of color represent the expression levels of DE microRNAs targeting host genes, where green represents a target of a down-regulated microRNA and red represents a target of up-regulated microRNA. A summary of KEGG enrichment analysis is found in S5 Table.

Moreover, identified KEGG pathways were not specifically significantly enriched for immune response signalling pathways. However, the top target genes of DE microRNAs were associated with endocytosis (IBV Mass41 3 h, IBV Mass41 18 h, and IBV DMV/1639 18 h groups), apoptosis (IBV DMV/1639 3 h and IBV Mass41 3 h groups) and mitogen-activated protein kinase (MAPK) (IBV Mass41 3 h and IBV DMV/1639 groups) signalling pathways. KEGG pathway enrichment for targets of DE microRNAs are shown in MAPK signalling pathway is shown in Fig 5.

### MicroRNA expression profiles in tracheas from IBV DMV/1639- and IBV Mass41-infected chickens

**Fig 6:**
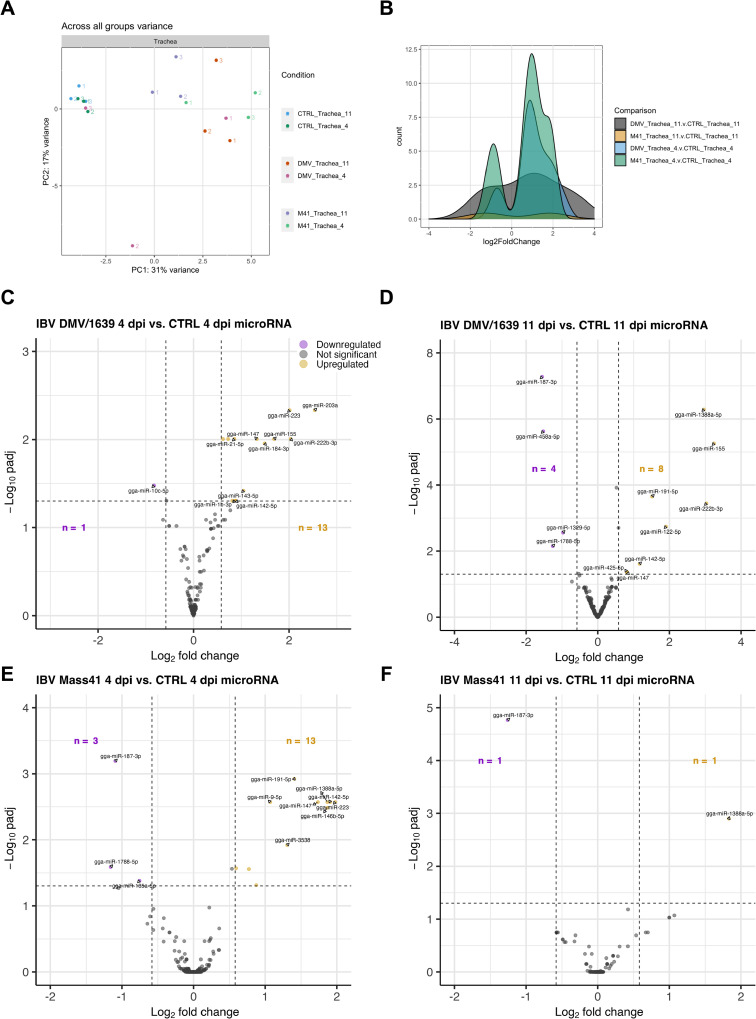
MicroRNA expression in tracheal tissues from chickens infected with IBV DMV/1639 or IBV Mass41. Panel (A) shows the variance across all samples based on the log counts of all microRNAs, while panel (B) represents the log_2_FC distribution of fluorescence signal intensity ratios for DE microRNAs in tracheal tissues from chickens infected with IBV DMV/1639 or IBV Mass41 at 4 dpi and 11 dpi in a histogram. The volcano plots present DE microRNAs in tracheal tissues from chickens infected with IBV DMV/1639 at 4 (A) and 11 dpi (B) or IBV Mass41 at 4 (C) and 11 dpi (D) relative to the respective control groups. The horizontal dotted line marks the threshold for the adjusted p-value <  0.05 and the vertical dotted lines denote the log2FC ≥  | 0.58 | (FC ≥  | 1.5|) threshold. Down-regulated microRNAs are indicated by purple data points and up-regulated microRNAs are indicated by yellow data points. A summary of all up- and down-regulated microRNAs for each treatment group are provided in Table 2.

Differential expression results for tracheal tissues from chickens infected with a high dose, i.e. 10^5^ EID_50_/bird, of IBV DMV/1639 or IBV Mass41, collected at 4 dpi and 11 dpi, are compiled in S6 Table. Table 2 contains results filtered for significantly differentially expressed microRNAs (defined by an adjusted p-value <  0.05 and a log_2_FC ≥  | 0.58|). The variance in log counts across all samples by group is shown in Fig 6A. Fig 6B shows the distribution of the log counts. The heatmaps (S2 Fig) provided in the supplementary files demonstrate the relationships between samples from the infected treatment groups, relative to the respective control groups based on differences in microRNA normalized log counts.

**Table 2:** DE microRNAs from tracheal tissues from chickens infected with IBV DMV/1639 or IBV Mass41.

Treatment group		Mature MicroRNA	Log_2_FC	Adjusted P-value
**IBV DMV/1639 4 dpi**	Down-regulated (1)	gga-miR-10c-5p	-0.830	3.337E-02
	Up-regulated (13)	gga-miR-203a	2.538	4.659E-03
		gga-miR-222b-3p	2.034	9.870E-03
		gga-miR-223	2.013	4.659E-03
		gga-miR-155	1.688	9.870E-03
		gga-miR-184-3p	1.487	1.108E-02
		gga-miR-147	1.323	9.870E-03
		gga-miR-143-5p	1.046	3.811E-02
		gga-miR-142-5p	0.888	4.958E-02
		gga-miR-1b-3p	0.864	4.958E-02
		gga-miR-21-5p	0.854	9.870E-03
		gga-miR-1388a-5p	0.809	4.958E-02
		gga-miR-222a	0.722	9.870E-03
		gga-miR-221-3p	0.618	9.870E-03
**IBV DMV/1639 11 dpi**	Down-regulated (4)	gga-miR-1329-5p	-0.972	2.741E-03
		gga-miR-1788-5p	-1.256	7.067E-03
		gga-miR-458a-5p	-1.522	2.372E-06
		gga-miR-187-3p	-1.555	5.284E-08
	Up-regulated (8)	gga-miR-155	3.245	5.504E-06
		gga-miR-222b-3p	3.032	3.614E-04
		gga-miR-1388a-5p	2.959	5.188E-07
		gga-miR-122-5p	1.905	1.814E-03
		gga-miR-191-5p	1.515	2.224E-04
		gga-miR-142-5p	1.165	2.425E-02
		gga-miR-147	0.822	4.479E-02
		gga-miR-425-5p	0.808	4.243E-02
**IBV Mass41 4 dpi**	Down-regulated (3)	gga-miR-135a-5p	-0.756	4.170E-02
		gga-miR-187-3p	-1.093	6.422E-04
		gga-miR-1788-5p	-1.159	2.570E-02
	Up-regulated (13)	gga-miR-222b-3p	4.641	2.324E-04
		gga-miR-155	4.083	1.360E-06
		gga-miR-223	1.977	2.682E-03
		gga-miR-142-5p	1.909	2.682E-03
		gga-miR-146b-5p	1.867	3.275E-03
		gga-miR-1388a-5p	1.867	2.682E-03
		gga-miR-147	1.736	2.682E-03
		gga-miR-191-5p	1.408	1.194E-03
		gga-miR-3538	1.307	1.199E-02
		gga-miR-9-5p	1.070	2.682E-03
		gga-miR-7	0.870	4.860E-02
		gga-miR-21-5p	0.772	2.767E-02
		gga-miR-20b-5p	0.593	2.690E-02
**IBV Mass41 11 dpi**	Down-regulated (1)	gga-miR-187-3p	-1.257	1.722E-05
	Up-regulated (1)	gga-miR-1388a-5p	1.830	1.253E-03

Among all IBV-inoculated groups, a total of 27 DE microRNAs were identified (Table 2), with 6 down-regulated and 21 up-regulated microRNAs. Fig 6C–F shows the number of down- and up-regulated microRNAs in each group, with a total of 14 and 12 DE microRNAs, 1 and 4 down-regulated microRNAs, and 13 and 8 up-regulated microRNAs for the IBV DMV/1639 4 dpi and IBV DMV/1639 11 dpi groups, respectively. For the IBV Mass41-infected groups, a total of 16 and 2 DE microRNAs, 3 and 1 down-regulated microRNAs, and 13 and 1 up-regulated microRNAs for the 4 dpi and 11 dpi groups, respectively. There tends to be a higher number of DE microRNAs at the 4 dpi time point for both IBV strains.

**Fig 7:**
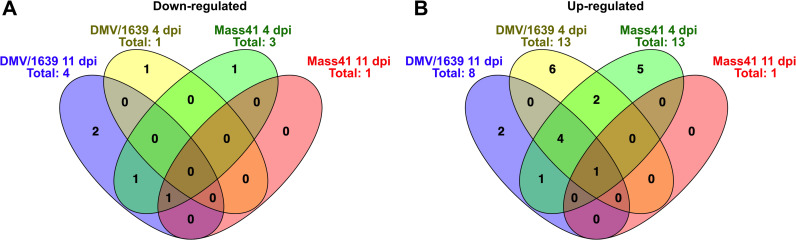
Overlap for DE microRNAs in tracheal tissues from chickens infected with IBV DMV/1639 or IBV Mass41. The overlap is shown by Venn diagrams for down-regulated (A) and up-regulated **(B)** DE microRNAs among tracheal tissues from chickens infected with IBV DMV/1639 or IBV Mass41 at 4 dpi and 11 dpi. The common and unique DE microRNAs are summarised in S8 Table.

Fig 7 shows the DE microRNA present in more than one treatment group for down-regulated microRNAs (Fig 7A) and for up-regulated microRNAs (Fig 7B). Details of the Venn diagram results can be found in S7 Table. No microRNAs were found to be commonly down-regulated among all groups. One microRNA in the IBV DMV/1639 11 dpi, IBV Mass41 11 dpi and IBV Mass41 4 dpi groups, and one microRNA in the IBV DMV/1639 11 dpi, IBV Mass41 4 dpi groups were commonly down-regulated. Furthermore, 1, 2, and 1 microRNAs were only down-regulated in the IBV DMV/1639 4 dpi, IBV DMV/1639 11 dpi, and IBV Mass41 4 dpi groups, respectively. For the up-regulated microRNAs, one was common among all treatment groups, 4 among the IBV DMV/1639 4 dpi, IBV DMV/1639 11 dpi, and IBV Mass41 4 dpi, 2 among the IBV DMV/1639 4 dpi and IBV Mass41 4 dpi groups, and 1 among the IBV DMV/1639 11 dpi and IBV Mass41 4 dpi groups. Finally, 6, 2, and 5 microRNAs were only up-regulated in the IBV DMV/1639 4 dpi, IBV DMV/1639 11 dpi, and IBV Mass41 4 dpi groups, respectively.

The microRNA profiles from tracheal samples show that the expression of DE microRNA is dependent on the strain and time point of collection, as only a few microRNAs were DE in more than one treatment group. Similar to the microRNA expression data from cTECs, no microRNAs are exclusively shared between the same IBV strain at different time points. The gga-miR-187-3p microRNA was down-regulated in the IBV DMV/1639 11 dpi, IBV Mass41 11 dpi and IBV Mass41 4 dpi groups. MicroRNAs gga-miR-155, gga-miR-222b-3p, gga-miR-147, and gga-miR-142-5p were up-regulated in the IBV DMV/1639 4 dpi, IBV DMV/1639 11 dpi, and IBV Mass41 4 dpi groups.

Upon comparing the DE microRNAs between each strain, it was shown that gga-miR-1788-5p was down-regulated for the IBV DMV/1639 11 dpi and IBV Mass41 4 dpi groups. MicroRNAs gga-miR-223 and gga-miR-21-5p were up-regulated in the IBV DMV/1639 4 dpi and IBV Mass41 4 dpi groups, while gga-miR-191-5p was up-regulated in the IBV DMV/1639 11 dpi and IBV Mass41 4 dpi groups. Finally, gga-miR-10c-5p in the IBV DMV/1639 4 dpi group, gga-miR-1329-5p and gga-miR-458a-5p in the IBV DMV/1639 11 dpi group, and gga-miR-135a-5p in the IBV Mass41 4 dpi group, were down-regulated only in those groups. MicroRNAs gga-miR-184-3p, gga-miR-221-3p, gga-miR-203a, gga-miR-222a, gga-miR-1b-3p, and gga-miR-143-5p in the IBV DMV/1639 4 dpi group, gga-miR-122-5p and gga-miR-425-5p in the IBV DMV/1639 11 dpi group, and gga-miR-3538, gga-miR-146b-5p, gga-miR-7, gga-miR-20b-5p, and gga-miR-9-5p in the IBV Mass41 4 dpi group, were up-regulated specifically in those groups.

Lastly, the miRDeep2 software predicted 63 novel microRNAs with miRDeep2 score >  4. The details for the miRDeep2 analysis for novel microRNAs in the cTEC small RNA-seq data are given in S8 Table.

### Target genes of DE microRNAs from the trachea of IBV-infected chickens

**Fig 8:**
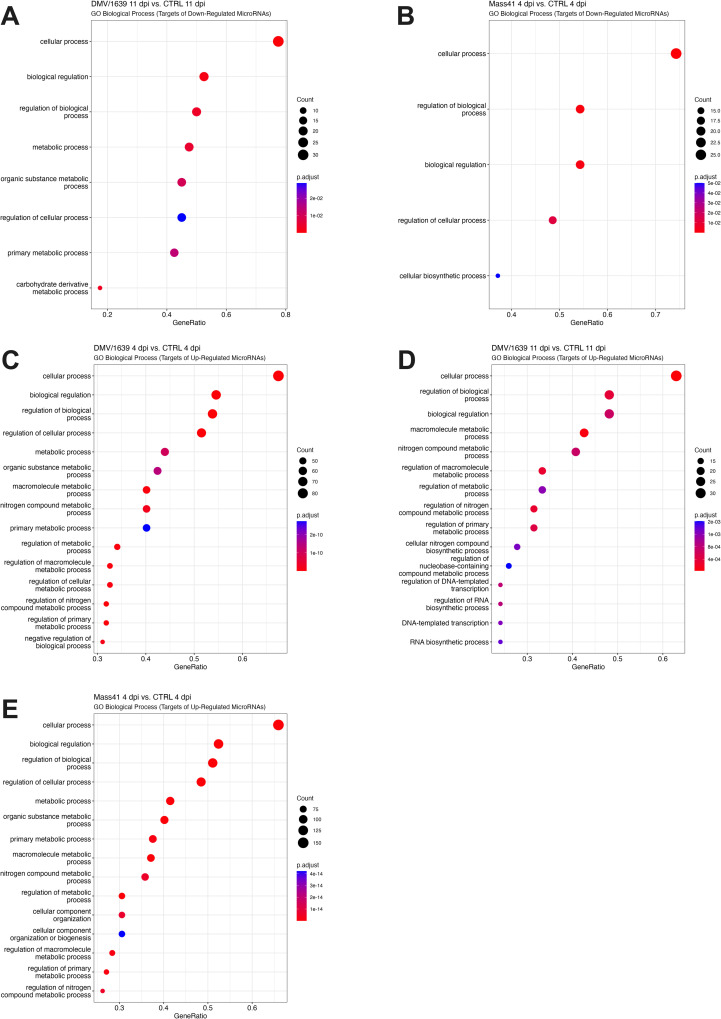
Functional analysis for targets of microRNAs in tracheal tissues from chickens infected with IBV DMV/1639 or IBV Mass41. The dot plots present the enriched GO (BP) terms for targets of down-regulated microRNAs of IBV DMV/1639 11 dpi (A) and IBV Mass41 4 dpi **(B)**, and for targets of up-regulated microRNAs of IBV DMV/1639 4 dpi (C) and 11 dpi **(D)**, and IBV Mass41 4 dpi (E) groups. Here, the “Count” indicates the number of genes enriched in a GO term, while the “GeneRatio” reflects the percentage of total target of DE microRNAs in a specific GO term. The intensity of color corresponds to the adjusted p-values. A summary of all GO terms for targets of DE microRNAs can be found in S9 Table.

Target gene prediction of microRNAs by miRDB [74,75] revealed at least 50 targets per group, excluding the IBV Mass41 11 dpi group, for the up-regulated microRNAs with a score of > 95 (S9 Table). No microRNA targets were identified for the IBV Mass41 11 dpi group and no significant enrichment for GO terms were found for the down-regulated target genes in the IBV DMV/1639 group. The absence of identified targets is due to the lower number of DE microRNAs and to the lack of predicted targets in miRDB for certain microRNAs. TRAF3 and TANK were both identified as predicted targets of gga-miR-155, a microRNA that was up-regulated in the IBV DMV/1639 4 dpi, IBV DMV/1639 11 dpi, and IBV Mass41 4 dpi groups. The enriched GO terms (Biological Process) for the top targets of DE microRNAs are shown in Fig 8. Full details for GO and KEGG enrichment analysis are given in S10 Table. As was the case with the microRNA targets from the cTECs, the top GO terms associated with the up- and down-regulated microRNA targets tend to be more associated with response to stimulus and metabolism.

**Fig 9:**
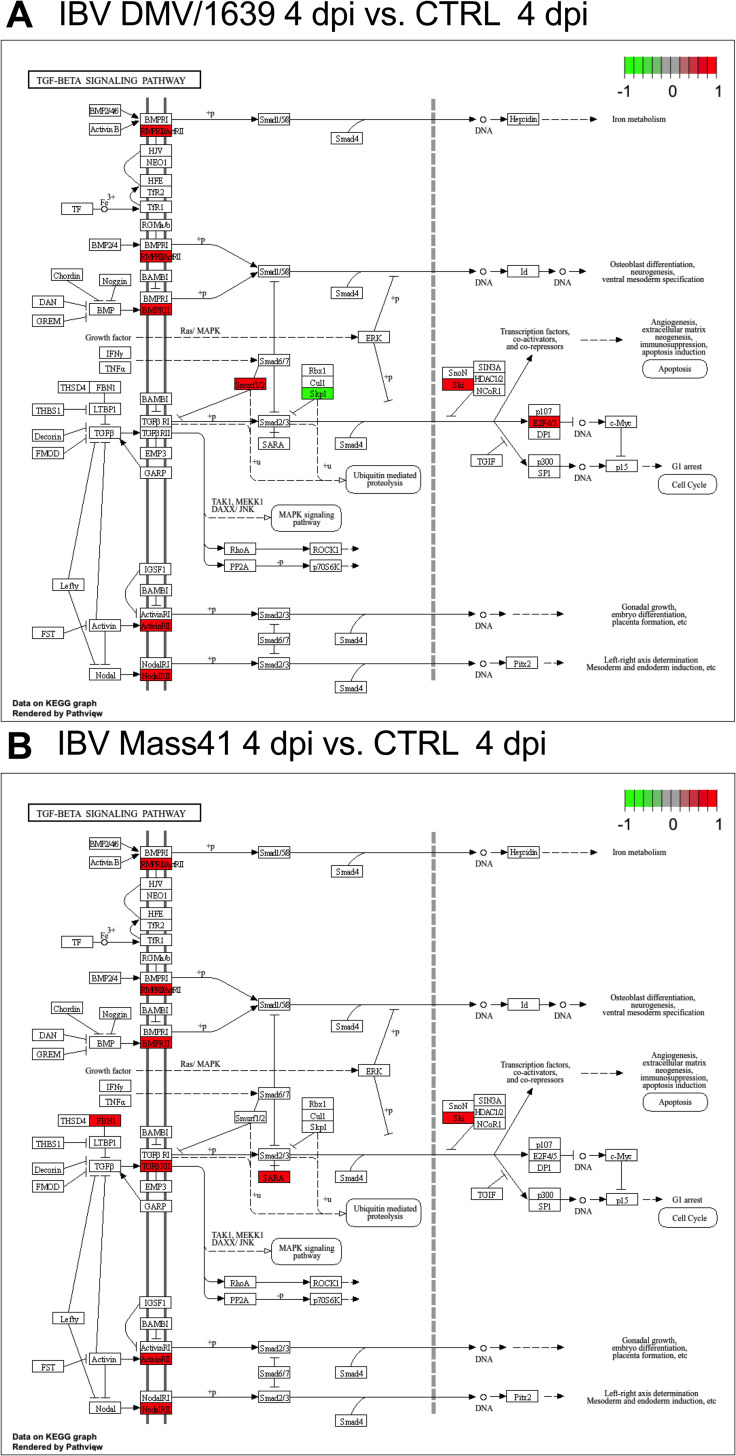
Pathway analysis for targets of DE microRNAs in tracheal tissues from chickens infected with IBV DMV/1639 or IBV Mass41. Pathway enrichment for IBV DMV/1639 4 dpi (A) and IBV Mass41 4 dpi **(B)** TGFβ signalling is illustrated. KEGG pathway analysis figures were generated using the R package *pathview* and the intensity of color represents the expression levels of DE microRNAs targeting host genes, where green represents a target of a down-regulated microRNA and red represents a target of up-regulated microRNA. Full details for KEGG enrichment analysis are found in S10 Table.

On the other hand, KEGG pathway enrichment analysis revealed that target genes from the DE microRNAs in the IBV DMV/1639 4 dpi and IBV Mass41 4 dpi groups were associated with endocytosis and TGFβ signaling pathways. TGFBβ signalling KEGG pathway enrichment is shown in Fig 9. Furthermore, the target genes from the IBV Mass41 4 dpi group were also associated with the MAPK signaling pathway. Finally, the target genes from IBV DMV/1639 4 dpi group were associated with the nucleotide oligomerization domain (NOD)-like receptor (NLR) and retinoic acid-inducible gene I (RIG-I)-like receptor (RLR) signaling pathways.

### Difference in DE microRNAs for the *in vitro* and *in vivo* infection models

**Fig 10:**
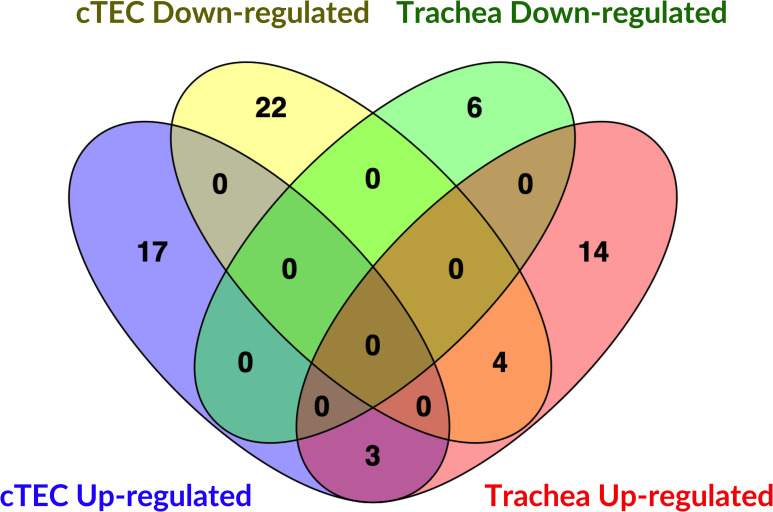
Overlap of DE microRNAs from cTECs and the trachea in the context of IBV DMV/1639 and IBV Mass41 infection. The overlap of DE microRNAs is illustrated by the Venn diagrams. The common and unique down-regulated and up-regulated DE microRNAs among cTECs and the trachea are shown and a summary of these comparisons are summarised in S11 Table.

The microRNA expression patterns of *in vitro* and *in vivo* small RNA-seq datasets varied significantly. Only 7 DE microRNAs were found to be common to both infection models in at least one treatment group (Fig 10 and S11 Table). MicroRNAs gga-miR-191-5p, gga-miR-425-5p, and gga-miR-222b-3p were up-regulated in cTECs and tracheas. Interestingly, gga-miR-155, gga-miR-184-3p, gga-miR-3538, and gga-miR-147 were down-regulated in cTEC and up-regulated in tracheas. Although we observed some overlaps in microRNA expression among the two infection models, the majority of microRNAs were unique to the *in vitro* or *in vivo* samples. Twenty-two down-regulated microRNAs and 17 up-regulated microRNAs were identified in the IBV-infected cTECs only. On the other hand, we observed 6 down-regulated microRNAs, including gga-miR-1329-5p, gga-miR-187-3p and gga-miR-458a-5p, and 14 up-regulated microRNAs, including gga-miR-146b-5p, gga-miR-7, gga-miR-21-5p, gga-miR-20b-5p, gga-miR-1388a-5p, in the IBV-infected tracheal tissues only.

### Host microRNA expression validation

The small RNA-seq results were validated by performing qPCR to detect expression of selected DE microRNAs using tracheal samples from IBV DMV/1639-infected chickens. The expression of two down-regulated and two up-regulated microRNAs were evaluated (Table 3). The deregulation of gga-miR-187-3p and gga-miR-458a-5p expression for the IBV DMV/1639 4 dpi group is not significant (log_2_FC ≥  | 0.58|) in both the small RNA-seq and qPCR results. The microRNA expression patterns from the qPCR experiments support the findings presented in this study as they are similar to the results obtained from small RNA-seq experiments in terms of magnitude of expression.

**Table 3:** Host microRNA expression fold-changes (FC) of selected microRNAs for small RNA-seq and qPCR results in tracheas from IBV DMV/1639-infected chickens relative to the uninfected control group at 4 dpi and 11 dpi.

		IBV DMV/1639 4 dpi		IBV DMV/1639 11 dpi	
	MicroRNA	Small RNA-Seq Log_2_FC	qPCR Log_2_FC	Small RNA-Seq Log_2_FC	qPCR Log_2_FC
**Down-regulated**	gga-miR-187-3p	-0.358	0.488	-1.555	-1.389
	gga-miR-458a-5p	-0.567	0.436	-1.522	-1.362
**Up-regulated**	gga-miR-1388a-5p	0.809	0.692	2.959	1.582
	gga-miR-155	1.688	2.245	3.245	2.999

### Chicken host microRNAs are predicted to target the IBV genome

**Fig 11:**
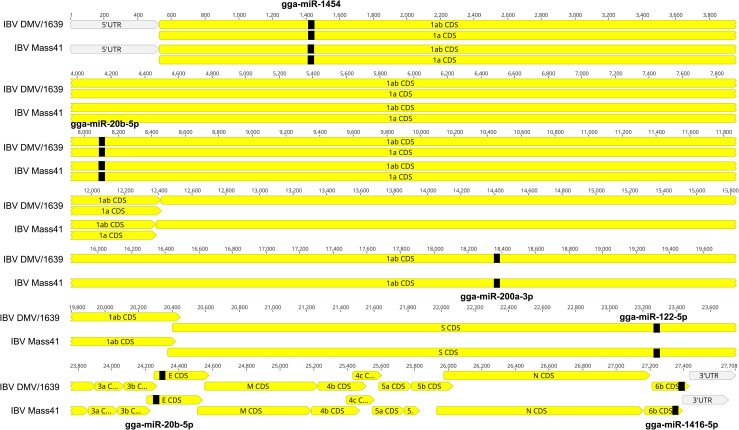
Chicken host microRNA predicted targets in the IBV genome. The target sites for microRNAs in the IBV genome, IBV DMV/1639 and IBV Mass41 were predicted using the miRanda, miRDB and RNAhybrid algorithms.

The top hits for the DE microRNAs targeting the IBV genome for both IBV DMV/1639 and IBV Mass41 strains are shown in Fig 11. Details for the targets considering all chicken microRNAs are given in S12 Table. The top hits for DE microRNA identified in this study targeted the viral RNA of both IBV DMV/1639 and IBV Mass41. Briefly, the identified microRNAs were gga-miR-1454 targeting the 1a gene, gga-miR-200a-3p and gga-miR-20b-5p targeting the 1ab gene, gga-miR122-5p targeting the S gene, gga-miR-20b-5p targeting the E gene and gga-miR-1416-5p targeting the 6b gene.

## Discussion

Viral infections, including those caused by IBV, continue to be one of the biggest bird health and economic threats to the global poultry industry. Gaining insight into the various factors that can influence the fundamental processes of IBV pathogenesis, especially at the primary site of infection, is crucial for devising novel approaches for control of IBV infections in chickens. For this reason, microRNA regulation in the context of viral infection presents an important avenue of research. In the present study, we aimed to characterize the profile of the cellular microRNAs following IBV infection *in vitro* and *in vivo*. We predicted that infection of chicken tracheal cells or tissues with IBV influences the host microRNA expression profiles and that some of these DE microRNA may have specific functions in the host response during infection. First, we demonstrated strain, time, and infection model-dependent changes in host microRNA expression. Second, we discovered important microRNAs in the context of IBV infection of the chicken trachea as gga-miR-155, gga-miR-1388a, gga-miR-7/7b, and gga-miR-21-5p, based on expression patterns and target features, and these could be utilized in future investigation to determine their roles in IBV infection control. Third, we determined that the *in vitro* and *in vivo* IBV infection models varied greatly in microRNA expression profiles, with 6 up-regulated and 17 down-regulated microRNAs expressed uniquely in the tracheal samples. Fourth, GO and KEGG pathway analysis revealed that the DE microRNAs can target genes in pathways such as the MAPK (cTECs) and TGFβ (trachea) signalling pathways. Finally, we predicted that the identified DE microRNAs can potentially interact with the IBV viral RNA, namely, gga-miR-1454 targeting the 1a gene, gga-miR-200a-3p and gga-miR-20b-5p targeting the 1ab gene, gga-miR122-5p targeting the S gene, gga-miR-20b-5p targeting the E gene and gga-miR-1416-5p targeting the 6b gene.

Our results show that there are a limited number of common DE microRNAs for the cTECs and trachea between the time points for the respective strains. This is somewhat expected and it may indicate a shift in the antiviral state of the host at the early time point or peak of infection [89,90] compared to at a later time point. Similarly, *Kemp et al*. observed no DE microRNAs at 48 hpi, but 17 DE microRNAs in the spleen and 7 DE microRNAs at 72 hpi in the lungs of IBV Mass41-infected embryos [36]. In the cTEC treatment groups, we observed a high degree of similarity between both strains at the same time point, with these groups sharing the majority of the DE microRNAs identified. Furthermore, the largest overlapping group of DE microRNAs was between the IBV DMV/1639 18 h and IBV Mass41 18 h groups, suggesting that these microRNAs may play similar roles during infection with either strain. For the tracheal tissues, the microRNA expression patterns are also consistent with a lack of common DE microRNAs between the 4 dpi and 11 dpi time points. On the other hand, while these viruses are similar in many ways, certain strain-specific differences in pathogenicity [91] may be responsible for the distinct microRNA expression patterns observed *in vivo*. It is important to note that the differences in DE microRNAs among the different virus strain groups at the same time points may also be due to the slightly different replication dynamics of each strain. For future experiments, evaluating more time points or using a dual RNA-seq approach to measure host (mRNA and microRNA) and viral transcripts from the same samples would provide a means of normalizing host differential gene and microRNA expression to the viral load.

It is known that microRNAs play key roles in regulating the immune responses by either activating or inhibiting critical immune response genes [92,93], and this has been shown in chickens [30,31]. We reported that the DE microRNAs had target genes involved in several different pathways, including the TGFβ and MAPK signaling pathways. TGFβ is a secreted cytokine and TGFβ signalling plays an important role in immunosuppression [94]. Recently, we demonstrated an increased expression of TGFβ in the bursa of Fabricius of IBV-infected chickens indicating potential immunosuppression in this organ [95]. While this study focuses on the trachea, the presence of specific microRNAs, gga-miR-21-5p, gga-miR-223 and gga-miR-203a, for example, with targets in this pathway indicates there may be a role for TGFβ signalling during IBV infection in the trachea. MAPK signalling has been shown to be involved during IBV infection [96–98]. Given the predicted target genes involved in this pathway, gga-miR-200a-3p, gga-miR-20b-5p and gga-miR-9-5p may modulate the course of infection. In addition, gga-miR-200a-3p was shown to regulate the immune response through MAPK signaling pathway in HD11 cells in the context of necrotic enteritis in chickens [99], suggesting that the effect of this microRNA may have an impact on a wide range of avian pathogens.

Although studies to compare changes in the tracheal microRNA expression profiles during IBV infection are not available, we can compare our findings with studies in other tissues. A previous study looking at IBV infection in chicken kidneys showed 7 highly differentially expressed microRNAs (gga-miR-30d, gga-miR-1454, gga-miR-7b, gga-miR-215-5p, gga-miR-1a-3p, gga-miR-3538 and gga-miR-2954) [33]. In our study, we confirmed the presence of some of these microRNAs during IBV infection. MicroRNAs gga-miR-1454 and gga-miR-7b were down-regulated in cTECs, gga-miR-2954 was up-regulated in cTECs, and gga-miR-3538 was up-regulated in trachea. Furthermore, embryonic spleen and lung from embryos infected with IBV Mass41 [36] presented several DE microRNA which were also present in our data set, such as gga-miR-1788-3p gga-miR-203a, gga-miR-200a-3p gga-miR-200b-3p, gga-miR-1b-3p, gga-miR-133c-3p, gga-miR-133a-3p, gga-miR-133b, and gga-miR-206. In addition, gga-miR-21-5p, which was up-regulated in the trachea for the IBV DMV/1639 4 dpi and IBV Mass41 4 dpi groups, was shown to inhibit antigen of avian dendritic cells upon being induced by IBV nsp7 and nsp16 [35].

We observed downregulation of gga-miR-133b following IBV infection of cTECs. Given the nature of the gga-miR-133 family of microRNA’s role in embryo myogenesis in chickens [100], it is expected that this microRNA is down-regulated. Furthermore, it was shown that gga-miR-133a-3p can significantly promote the upregulation of differentiation-related muscle-derived factors, in turn promoting the differentiation of myoblasts. Another microRNA that plays a role in myogenesis in chickens is gga-miR-206 [101] and it was down-regulated at both time points for IBV DMV/1639-infected cTECs and early time point in IBV Mass41 infected cTECs.

MiR-155 is an important and well described microRNA across many species with various different functions, such as immune cell regulation [102]. MicroRNA gga-miR-155 was down-regulated in the cTEC IBV Mass41 18 h group, and up-regulated in the tracheal IBV DMV/1639 4 dpi, IBV DMV/1639 11 dpi, and IBV Mass41 4 dpi groups. This microRNA has known roles in immune response of chickens, such as enhancing type I IFNs to supress IBDV replication [24] and as a regulator of the immune response to different vaccines through the MAPK and mTOR signalling pathways [103]. Taken together, gga-miR-155 may play a role during IBV infection.

It is known that viral infections cause a shift in the host microRNA expression profile [18]. Some microRNAs may inhibit viral replication or promote the host antiviral responses, while others may have the opposite effect and promote viral replication or inhibit host antiviral responses. We reported that the presence of microRNA with the potential to directly interact with different parts of the IBV genome, indicating that they may modulate viral replication. One example of a host microRNA interaction directly with a virus is miR-29a, which restricts viral replication of human immunodeficiency virus (HIV)-1 by binding to its 3’UTR [104]. On the other hand, miR-146a was shown to enhance viral replication of influenza A virus by inhibiting type I IFN production [105]. As a result, evaluating the microRNA expression profiles can only inform us about the shifts in expression patterns and not about the ultimate function during infection. Target prediction and functional analysis help in providing insight into the potential roles of microRNAs.

While *in vitro* and *ex vivo* systems can provide a platform for studying mechanisms of infection under highly controlled conditions, the results and phenomena observed may vary when compared to *in vivo* infection experiments. For example, Reemers *et al.* evaluated the early immune responses to AIV infection in tracheal organ culture (TOC) and in *in vivo* infected trachea [106]. They found that the models shared only a small number of genes that were differentially expressed. Just as host cells and tissues express different protein-coding genes, they express different microRNAs and this may be contributing to the difference in expression profiles. Chen *et al.* demonstrated that the microRNA expression of airway epithelial cells cultured at an air-liquid interface is inconsistent with the *in vivo* expression patterns, which they account to not being able to replicate the *in vivo* environment, which would have an impact of the microRNA expression [107]. In another study looking a microRNAs in mature glia and cultured cells, it was shown that most microRNAs decreased after cells were cultured and that some of the microRNAs that were not present *in vivo* had increased expression *in vitro* [108]. Ultimately, the changes or differences in *in vitro* and *in vivo* models may be due to adaptation to cell culture conditions and should be considered when inferring microRNA patterns and behaviours from *in vitro* studies.

Finally, the limited available information on functionally validated chicken microRNAs and the associated microRNA targets and gene ontology terms, which are occasionally inferred from studies in humans and mice, presents an important limitation for microRNA studies in chickens. With an increase in interest and need for microRNA research in poultry, the quantity and quality of data extracted from these types of experiments will improve. The ability to compare between microRNA studies in the context of IBV infection in chickens reveals interesting similarities and patterns, but the reliability of these connections is limited by the differences in animals or cell lines used and their experimental conditions, the different virus strains used, as well as the different RNA isolation and sequencing methods.

## Conclusions

In conclusion, the characterization of small regulatory RNA transcriptome data reveals novel players in IBV pathogenesis. Comparison between different IBV strains, time points of collection and *in vitro* and *in vivo* models of infection provided a stronger and more supported interpretation of microRNA expression and regulation in the context of IBV infection. Our results allow us to describe the changing microRNA profiles over the course of infection. We identified a total of 8, 32, 8 and 34 DE microRNAs for the IBV DMV/1639 3 h, IBV DMV/1639 18 h, IBV Mass41 3 h and IBV Mass41 18 h groups, respectively, and a total of 14, 12, 16 and 2 DE microRNAs for the IBV DMV/1639 4 dpi, IBV DMV/1639 11 dpi, IBV Mass41 4 dpi and IBV Mass41 11 dpi groups, respectively.

We reported, for the first time, the tracheal profiles of microRNAs involved in IBV infection with different strains of IBV, and that these specific microRNAs may modulate the antiviral response and the viral replication upon IBV infection in chickens. We identified candidate microRNAs, including gga-miR-155, gga-miR-1388a, gga-miR-7/7b, and gga-miR-21-5p. The results of this study, in conjunction with our previously published mRNA expression datasets [37], provide insight into the details of host-virus interactions during IBV infection in chickens; information which is key for designing effective control strategies against this pathogen. These insights open up avenues for future work developing targeted strategies to improve poultry health. Future studies will confirm the direct effects or regulatory functions of candidate microRNAs on the induction of the antiviral response and replication of IBV *in vitro* and *in vivo*.

## Supporting information

S1 Fig cTEC microRNA heatmaps.
(TIF)

S2 Fig Trachea microRNA heatmaps.
(TIF)

S1 Table All DEGs microRNA cTEC.
(XLSX)

S2 Table Venn diagrams microRNA cTEC overlap.
(XLSX)

S3 Table Novel microRNA miRDeep2 cTEC.
(XLSX)

S4 Table cTEC target genes miRDB.
(XLSX)

S5 Table cTEC microRNA targets GO and KEGG.
(XLSX)

S6 Table All DEGs microRNA trachea.
(XLSX)

S7 Table Venn diagrams microRNA trachea overlap.
(XLSX)

S8 Table Novel microRNA miRDeep2 trachea.
(XLSX)

S9 Table Trachea target genes miRDB.
(XLSX)

S10 Table Trachea microRNA targets GO and KEGG.
(XLSX)

S11 Table Venn diagrams microRNA cTEC trachea overlap.
(XLSX)

S12 Table Viral targets microRNA miRanda and miRDB cTEC trachea.
(XLSX)
