# Supplementary figures and images for: Characterization of microRNA candidates at the primary site of infectious bronchitis virus infection: A comparative study of in vitro and in vivo avian models
Source: PLoS One. 2025 Mar 11;20(3):e0319153. doi: 10.1371/journal.pone.0319153 (PMC11896067; doi:10.1371/journal.pone.0319153)

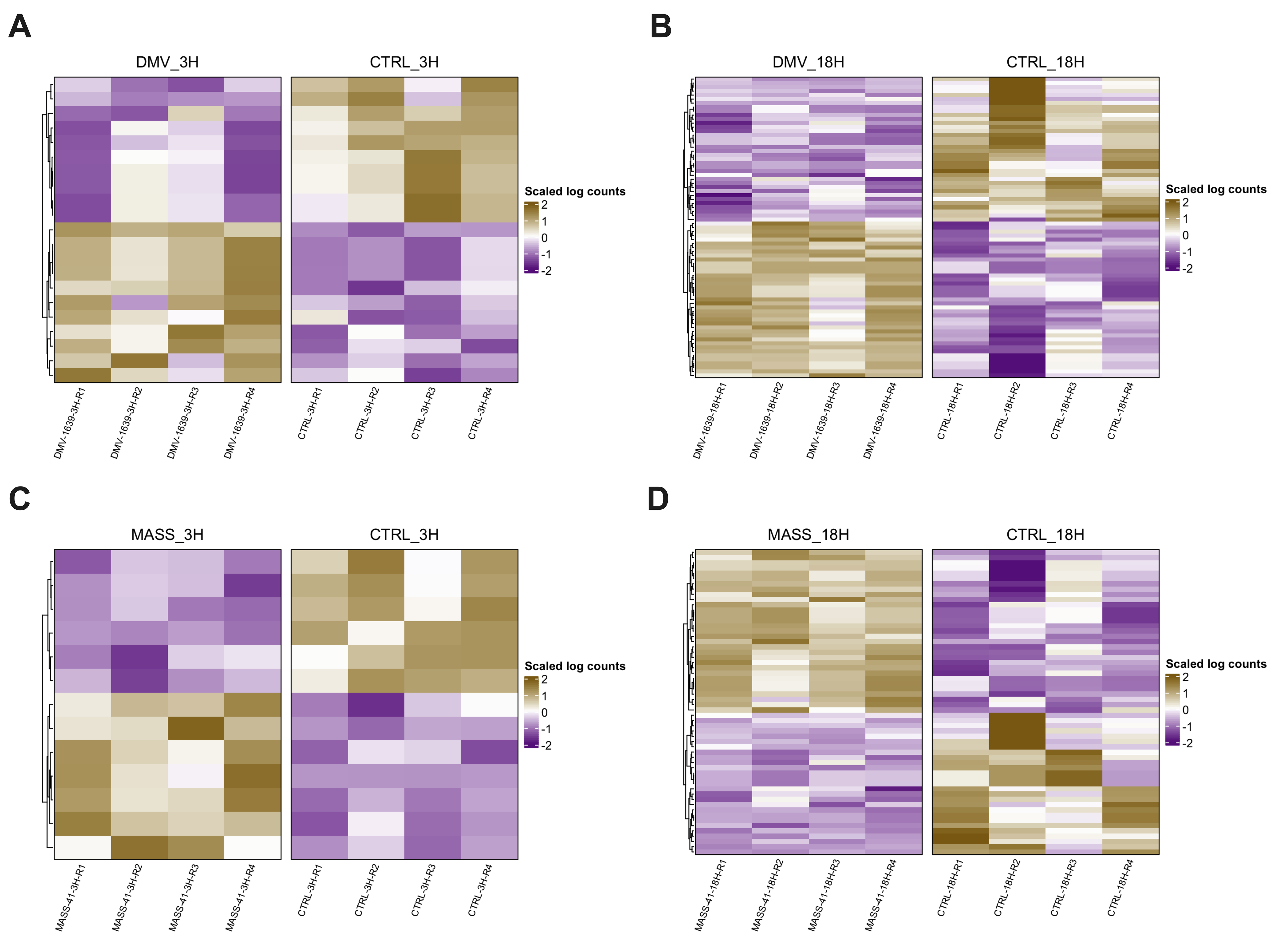

Supplement: S1 Fig — (TIF) [file pone.0319153.s001.tif]

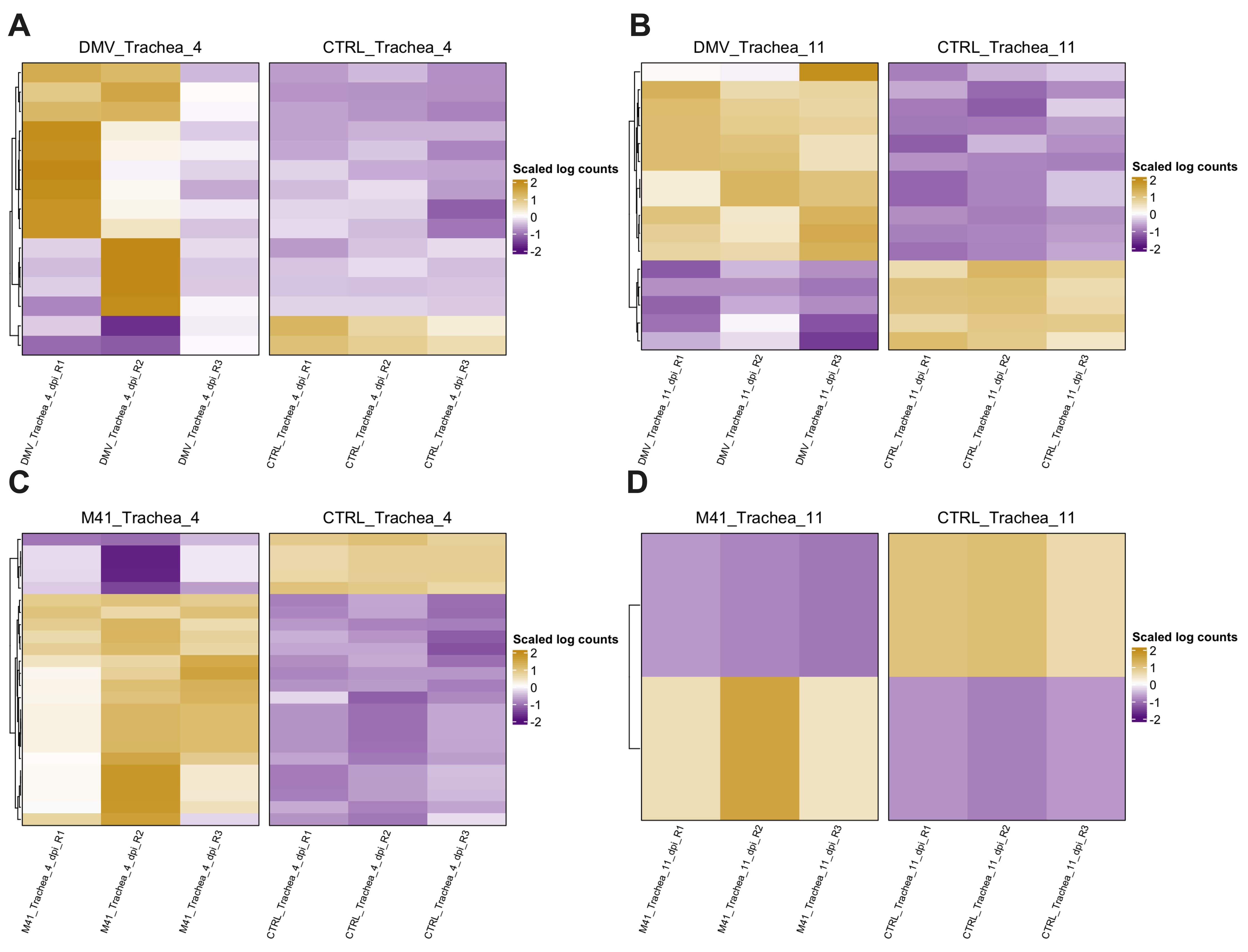

Supplement: S2 Fig — (TIF) [file pone.0319153.s002.tif]
